# Analysis of clinical factors affecting corneal endothelial cell density in eye bank donors

**DOI:** 10.3389/fopht.2026.1952825

**Published:** 2026-09-16

**Authors:** Lan Yu, Wei Ming, Shangcao Wu, Qiaoqiao Dong, Jingwei Li, Jiao Qin, Qingyan Zeng

**Affiliations:** 1 Aier Eye Hospital of Wuhan University, Wuhan, Hubei, China; 2 Wuhan Red Cross Aier Eye Bank, Wuhan, Hubei, China; 3 Wuhan Aier Hankou Eye Hospital, Wuhan, Hubei, China

**Keywords:** corneal endothelial cell density, corneal transplantation, donor cornea, eye bank, influencing factors, linear mixed model

## Abstract

**Purpose:**

To systematically analyze factors influencing corneal endothelial cell density (ECD) in eye bank donors and provide robust evidence for optimizing donor screening strategies.

**Methods:**

A retrospective analysis was conducted on 1438 corneas from 782 donors (2013–2025). Associations between ECD and donor age, sex, laterality, cause and season of death, cataract surgery, cancer, Diabetes Mellitus (DM), and death-to-preservation time (DTP) were evaluated using univariate analysis and a generalized linear mixed model (GLMM).

**Results:**

Univariate analysis showed ECD was significantly associated with age, cataract surgery, DTP, cause of death, and death season (all *P* < 0.05). The GLMM indicated that age, cataract surgery, and DTP were highly significant independent predictors (all *P* < 0.001): ECD was 287.737 cells/mm² higher in donors without cataract surgery (95% CI: 177.109–398.365), and decreased by 3.847 cells/mm² (95% CI: -5.561 to -2.134) per year of age and 18.342 cells/mm² (95% CI: -27.623 to -9.062) per hour increase in preservation time. Among causes of death, only the cardiovascular/cerebrovascular disease group differed significantly from the chronic organ failure group (*P* = 0.013); other factors including sex, eye laterality, cancer, DM, and death season showed no independent effects (all *P*>0.05).

**Conclusion:**

Advanced age, prior cataract surgery, and prolonged death-to-preservation time were independently associated with lower ECD. Cause of death was not an independent predictor overall, although donors who died from CCVDs had significantly higher ECD than those with chronic organ failure. These findings provide a robust evidence base for clinical donor screening and allocation, thereby maximizing the utility of scarce donor corneas.

## 1 Introduction

Globally, approximately 2.9 million people experience visual impairment due to corneal diseases and require corneal transplantation surgery to restore visual function (1, 2). Donor cornea quality is a key factor affecting postoperative outcomes of corneal transplantation, with endothelial cell density (ECD) recognized as the most critical quality assessment parameter (3). As the number of patients with corneal blindness continues to rise, the global shortage of donor corneas has become increasingly prominent, forming the primary bottleneck restricting corneal transplantation surgeries. Statistics indicate that in developed countries such as Germany, over one-third of procured donor corneas are discarded due to failure to meet quality standards, with the discard rate being even higher in regions with limited resources (4).

Previous studies suggest that factors such as age and cataract surgery may affect ECD, yet most research suffers from limitations including small sample sizes, a narrow focus on specific influencing factors, and inadequate control for inter-eye correlations within the same donor (5, 6). Additionally, the impact of systemic diseases such as Diabetes Mellitus (DM) and cancer on ECD remains controversial. While some studies suggest DM may indirectly damage the corneal endothelium through microvascular complications, this effect was not statistically significant in multivariate analyses (7).

Using a large cohort of 1438 corneal donor samples, this study appliedunivariate analysis and Generalized Linear Mixed Models (GLMM) to control for nested effects in bilateral data from the same donor. We systematically evaluated the independent effects of multidimensional clinical factors on ECD, to provide more comprehensive and reliable evidence-based support for clinical donor screening.

## 2 Materials and methods

### 2.1 General information

This retrospective case series study collected and analyzed data regarding 1,438 corneas obtained from 782 human donors at the Wuhan Red Cross Aier Eye Bank between December 2013 and November 2025.

Inclusion criteria included: (1) Complete donor clinical data, including key information such as donor sex, age, cause of death, cataract surgery, cancer, DM, and death-to-cornea preservation time; (2) Complete slit-lamp microscopy and corneal endothelial examination data; and (3) Corneal tissue integrity, free from significant mechanical trauma, infection, corneal scarring, or congenital corneal pathologies.

Exclusion criteria included: (1) unknown cause of death; (2) infectious and communicable diseases, defined as individuals with positive serological tests for HIV, hepatitis B, or hepatitis C, or those with acute viral encephalitis, encephalitis/encephalopathy of unknown etiology, acute septicemia, or acute bacterial/fungal endocarditis; (3) malignancies, including ocular metastasis of malignant tumors, acute leukemia, or acute disseminated lymphoma; (4) local ocular diseases, such as ocular malignancies or acute infection of the eyeball or intraocular structures; (5) Corneas with severe stromal edema or prominent Descemet’s folds that preclude accurate endothelial microscopic evaluation were also excluded.

Cause of death was categorized into four groups: malignant tumors, cardiovascular-cerebrovascular diseases (CCVDs), chronic organ failure (a composite group consisting of end-stage renal, hepatic, respiratory and cardiac failure), and traumatic accidents.

### 2.2 Acquisition and preservation of donor corneas

Eye bank physicians performed all enucleations following standard protocols. The closed eyelids and periorbital skin were disinfected twice with 1% povidone-iodine. After applying a fenestrated drape and retracting the eyelids with a speculum, the conjunctival sac was irrigated with 5% povidone-iodine, then rinsed with 0.9% normal saline. Following a 360°conjunctival incision and dissection of the extraocular muscles and connective tissue, the optic nerve was severed and the globe enucleated. Each globe was placed in a labeled sterile container with sterile gauze moistened with saline, and transported at 2–8 °C to the eye bank. Slit lamp examination of the cornea confirmed absence of significant trauma, infection, scarring, or congenital abnormalities. The globe was rinsed with saline at 4 °C; after trimming conjunctiva, fascia, and extraocular muscles, it was immersed in povidone-iodine for 2 min, rinsed, then immersed in 1/1000 tobramycin saline for 15–20 min. Under a Class II Type A2 biosafety cabinet, an 18 mm corneal trephine was used to incise the sclera 3 mm posterior to the limbus. The circumferential cut was completed with corneal scissors, and the corneoscleral button (with minimal uveal tissue) was harvested using forceps. The button was stored in Eusol-C preservation solution (PAOLO GOBBI FRATTINI S.r.I, Milan, Italy, SCC003.20) at 4 °C for up to 14 days. The remaining sclera, after removal of all intraocular and pigmented tissues, was immersed in 75% alcohol for 24 h, then stored in 95% alcohol at 4 °C for up to 3 years. Death-to-preservation time (DTP), defined as the interval from donor death to corneal immersion in Eusol-C, may affect endothelial status during the post-mortem and pre-preservation period. All endothelial cell density (ECD) measurements were performed immediately after preservation under stable standardized conditions; the ultra-short detection time did not alter ECD values, methodologically distinguishing them from DTP.

### 2.3 Donor corneal endothelial cell assessment

Immediately after placing the donor cornea into the preservation solution, central endothelial cell images were captured using an eye bank specular microscope (HAI EB-3000xyz, USA). Endothelial cell counts were performed three times, and the average value was calculated and recorded.

### 2.4 Statistical methods

Statistical analyses were performed using IBM SPSS Statistics 26.0. Normality of continuous variables was assessed with the Shapiro–Wilk test. Normally distributed data were expressed as mean ± SD and compared using the independent-samples t-test (two groups) or one-way ANOVA with LSD post-hoc tests (multiple groups). Non-normally distributed data were presented as median (Q1, Q3) and compared using the Mann–Whitney U test (two groups) or Kruskal–Wallis H test (multiple groups), followed by Dunn’s test with Bonferroni correction. Categorical variables were summarized as frequencies and percentages.

To account for the correlation between paired eyes from the same donor, a GLMM with a Gaussian distribution and identity link was fitted, with donor ID included as a random intercept. Although raw corneal endothelial cell density (ECD) was not normally distributed, residual diagnostics (histogram and Q–Q plot) showed approximate normality, justifying this specification. Coefficients were therefore interpretable as absolute changes in ECD (cells/mm²). Satterthwaite approximation was used to calculate denominator degrees of freedom. Model building proceeded from a full-variable GLMM, with interaction testing and Akaike Information Criterion (AIC)-guided reduction yielding the final parsimonious model. All tests were two-tailed; P < 0.05 was considered statistically significant.

## 3 Results

### 3.1 Donor characteristics and corneal preservation status

The mean donor age was 66.19 ± 12.11 years (range: 6–91 years), comprising 998 males (69.4%) and 440 females (30.6%). Right eyes accounted for 724 cases (50.3%) and left eyes for 714 cases (49.7%). The causes of death included malignant tumors (654 cases; 45.5%), cardiovascular-cerebrovascular diseases (CCVDs) (409 cases; 28.4%), chronic organ failure (355 cases; 24.7%), and traumatic accidents (20 cases; 1.4%). The mean ECD was 2588.2 ± 326.04 cells/mm² (range: 939–3574 cells/mm²), and the mean death-to-preservation time (DTP) was 5.79 ± 2.16 h (range: 0.5-21.67 h). Detailed demographic and clinical characteristics are presented in **Tables 1** and **2**.

**Table 1:** Demographic and clinical characteristics of donors.

Variable	Group	Number of cases	Percentage (%)
Sex	Male	998	69.4
	Female	440	30.6
Age	≤30 years	17	1.2
	31–50 years	118	8.2
	51–60 years	244	17.0
	61–70 years	494	34.4
	71–80 years	424	29.5
	≥81 years	141	9.8
Eye laterality	Right eye	724	50.3
	Left eye	714	49.7
Cause of death	Malignant tumors	654	45.5
	CCVDs	409	28.4
	Chronic organ failure	355	24.7
	Traumatic accidents	20	1.4
Season of Death	Spring	357	24.8
	Summer	320	22.3
	Autumn	429	29.8
	Winter	332	23.1
Cataract surgery	No	1400	97.4
	Yes	38	2.6
Cancer	No	761	52.9
	Yes	677	47.1
DM	No	1259	87.6
	Yes	179	12.4

Data are presented as number and percentage. CCVDs, cardiovascular- cerebrovascular diseases; DM, Diabetes Mellitus. All data were collected from 1438 corneas of 782 eye bank donors (2013–2025).

**Table 2:** Descriptive statistics of core donor indicators.

Variable	Mean	Range	Median
Age (years)	66.19 ± 12.11	6-91	67
DTP (h)	5.79 ± 2.16	0.5-21.67	5.33
ECD (cells/mm²)	2588.20 ± 326.04	939-3574	2627.50

Data are presented as mean ± standard deviation (SD), range, and median. DTP, death-topreservation time; ECD, corneal endothelial cell density. Shapiro-Wilk test indicated ECD, age, and DTP were non-normally distributed (all *P* < 0.001).

### 3.2 Correlation analysis between donor characteristics and donor corneal ECD

The Shapiro-Wilk test results indicated that ECD, age, and DTP deviated significantly from a normal distribution (*P* < 0.001). Spearman correlation analysis showed that donor age (r=-0.118), cataract surgery (r=-0.104), and DTP (r=-0.173) exhibited significant negative correlations with ECD (all *P* < 0.001). Cancer (r=0.040, *P* = 0.129) and DM (r=-0.041, *P* = 0.120) did not demonstrate a statistically significant correlation with ECD (**Table 3**).

**Table 3:** Spearman correlation analysis between various clinical factors and ECD.

Variable	Spearman’s r	*P-*value
Age (years)	-0.118	<0.001
Cataract surgery	-0.104	<0.001
Cancer	0.040	0.129
DM	-0.041	0.120
DTP (h)	-0.173	<0.001

Spearman correlation analysis was used for non-normally distributed variables. ECD, corneal endothelial cell density; DM, Diabetes Mellitus; DTP, death-to-preservation time. *P* < 0.05 was considered statistically significant.

The Mann-Whitney U test and the Kruskal-Wallis H test showed that sex (Z=-0.945, *P* = 0.345), eye laterality (Z=-0.786, *P* = 0.432), cancer (Z=-1.516, *P* = 0.129), and DM (Z=-1.554, *P* = 0.120) had no significant correlation with ECD. Age (χ²=29.085, *P* < 0.001), cause of death (χ²=13.885, *P* = 0.003), season of death (χ²=10.621, *P* = 0.014), and cataract surgery (Z=-3.934, *P* < 0.001) were not significantly associated with ECD. Cataract surgery and age suggested the most significant associations (**Table 4**).

**Table 4:** Impact of different factors on donor ECD.

Variable	Group	ECD (cells/mm²) median	Test statistic (Z/χ²)	*P*-value
Sex	Male	2632	Z=-0.945	0.345
	Female	2621		
Eye laterality	Right eye	2631	Z=-0.786	0.432
	Left eye	2625		
Age	≤30 years	2929	*χ*^2^ = 29.085	<0.001
	31–50 years	2703		
	51–60 years	2632		
	61–70 years	2627		
	71–80 years	2630		
	≥81 years	2531		
Cause of death	CCVDs	2649	*χ*^2^ = 13.885	0.003
	Malignant tumors	2629.5		
	Traumatic accidents	2638		
	Chronic organ failure	2600		
Season of Death	Spring	2649	*χ*^2^ = 10.621	0.014
	Summer	2638.5		
	Autumn	2623		
	Winter	2604		
Cataract surgery	No	2631	Z=-3.934	<0.001
	Yes	2260.5		
Cancer	No	2627	Z=-1.516	0.129
	Yes	2631		
DM	No	2630	Z=-1.554	0.120
	Yes	2624		

Data are presented as median ECD (corneal endothelial cell density). Mann-Whitney *U* test (Z) was used for two-group comparisons, and Kruskal-Wallis H test (χ²) for multiple-group comparisons. CCVDs, cardiovascular-cerebrovascular diseases; DM, Diabetes Mellitus. *P* < 0.05 was considered statistically significant.

Dunn *post hoc* pairwise comparisons with Bonferroni correction revealed that, regarding age groups, the ≥81 years group had significantly lower ECD than the ≤30 years, 31–50 years, and 51–60 years groups; the 61–70 years and 71–80 years groups had significantly lower ECD than the ≤30 years group. Among causes of death, donors who died of chronic organ failure exhibited significantly lower ECD than those who died of malignant tumors or CCVDs; Regarding the season of death, ECD was significantly lower in winter than in spring (all Bonferroni-corrected *P* < 0.05); No statistically significant differences were observed in any remaining pairwise comparisons (all Bonferroni-corrected *P* ≥ 0.05) (**Table 5**).

**Table 5:** Results of Dunn’s *post hoc* pairwise comparisons for donor ECD-related multi-categorical variables.

Grouping type	Comparison groups	Dunn’s Z value	Bonferroni-corrected *P-*value
Age group	≥81 years vs. ≤30 years	4.033	0.001
	≥81 years vs. 31–50 years	4.295	0.000
	≥81 years vs. 51–60 years	3.165	0.023
	71–80 years vs. ≤30 years	3.074	0.032
	61–70 years vs. ≤30 years	3.069	0.032
Cause of death	Chronic organ failure vs malignant tumors	2.922	0.021
	Chronic organ failure vs CCVDs	3.441	0.003
Season of Death	Winter vs spring	2.985	0.017
	All other pairwise comparisons	–	>0.05

Dunn’s *post hoc* test with Bonferroni correction was performed for multiple pairwise comparisons after Kruskal-Wallis H test. ECD, corneal endothelial cell density; CCVDs, cardiovascular-cerebrovascular diseases. A Bonferroni-corrected *P* < 0.05 was considered statistically significant.

A GLMM including all potential influencing factors was constructed, treating donor ID as a random effect. Mean donor age stratified by cause-of-death is presented in **Supplementary Table S1**; the GLMM comparisons for cause-of-death were fully adjusted for donor age. In the full-variable GLMM, cancer showed borderline statistical significance (P = 0.045). We further tested interaction terms between cancer and other covariates; no interaction reached significance (all P>0.05). Model comparison using AIC supported the parsimonious reduced model excluding cancer together with sex, eye laterality, season of death and DM, which yielded lower AIC. These variables were thus removed from the final fixed-effects specification. The final parsimonious GLMM incorporated age, cause of death, cataract surgery, and DTP as fixed effects, demonstrating good overall model fit (F = 12.304, *P* < 0.001). Results showed that age (F = 19.402, *P* < 0.001), cataract surgery (F = 26.031, *P* < 0.001), and DTP (F = 15.032, *P* < 0.001) were highly significant independent predictors. The overall effect of cause of death was not significant (F = 2.109, *P* = 0.097), but ECD was significantly higher in donors who died from CCVDs compared to those with chronic organ failure (*P* = 0.013; **Table 6**).

**Table 6:** Fixed-effects analysis of variance (F-test) for the generalized linear mixed model (GLMM).

Parameter	F	df_1_	df_2_	*P*-value
Adjusted model	12.304	6	1431	0.000
Age	19.402	1	1431	0.000
Cause of death	2.109	3	1431	0.097
Cataract surgery	26.031	1	1431	0.000
DTP (h)	15.032	1	1431	0.000

GLMM was constructed with donor ID as a random effect to account for inter-eye correlations. F-test was used to assess the significance of fixed effects. DTP, death-to-preservation time. *P* < 0.05 was considered statistically significant.

Further analysis of effect sizes for core variables revealed that each 1-year increase in age was associated with an average ECD decrease of 3.847 cells/mm² (β=-3.847, 95% CI: -5.561 to -2.134, *P* < 0.001). Subjects without cataract surgery exhibited an average ECD 287.737 cells/mm² higher than those who underwent surgery (β=287.737, 95% CI: 177.109 to 398.365, *P* < 0.001). Each 1-hour increase in DTP was associated with an average ECD reduction of 18.342 cells/mm² (β=-18.342, 95% CI: -27.623 to -9.062, *P* < 0.001). Donors who died from CCVDs exhibited an average ECD that was 70.736 cells/mm² higher than those who died from chronic organ failure (β=70.736, 95% CI: 14.719 to 126.754, *P* = 0.013; **Table 7**).

**Table 7:** Regression coefficients and effect size analysis from the GLMM.

Parameter	Regression coefficient (β)	Standard error (SE)	t-value	95% CI		*P*-value
Intercept	2630.252	91.8149	28.647	2450.146	2810.359	0.000
Age	-3.847	0.8735	-4.405	-5.561	-2.134	0.000
Cause of death=0 (reference 3)	70.736	28.5569	2.477	14.719	126.754	0.013
Cause of death=1	30.986	26.3816	1.175	-20.765	82.737	0.240
Cause of death=2	59.14	91.2352	0.648	-119.829	238.109	0.517
Cataract surgery (reference 1)	287.737	56.3962	5.102	177.109	398.365	0.000
DTP (h)	-18.342	4.731	-3.877	-27.623	-9.062	0.000

GLMM, generalized linear mixed model; CI, confidence interval; DTP, death-to-preservation time. Reference for cause of death: 3, chronic organ failure (0, CCVDs, 1, malignant tumors, 2, traumatic accidents); reference for cataract surgery: 1, Yes (0, No). P < 0.05 was considered statistically significant.

## 4 Discussion

Corneal endothelial cells are key structures maintaining corneal transparency; ECD directly determines donor cornea quality and post-transplantation outcomes. Identifying its key influencing factors is crucial for optimizing donor allocation and reducing transplant failure risks (8, 9). Based on large-scale data from 1438 donor corneas, this study systematically evaluated the independent effects of a range of clinical factors on ECD using univariate analysis and GLMMs. The results revealed that donor age, cataract surgery, and DTP are key factors associated with ECD, while cause of death could serve as a secondary reference indicator. These findings are largely consistent with international eye bank research conclusions over the past five years (5, 10, 11).

Consistent with previous reports (5, 6, 12, 13), advanced age significantly predicted lower endothelial cell density (ECD). Corneal endothelial cells are terminally differentiated and do not proliferate after birth, with physiological age-related loss estimated at 0.3%–0.6% per year (14). In our cohort, ECD declined by 3.847 cells/mm² annually (~0.15% relative loss, based on a mean ECD of 2588.2 cells/mm²). This decline was less pronounced than the physiological loss observed in living individuals (13), which may be attributable to multivariate adjustment for prior cataract surgery—a condition disproportionately prevalent among elderly donors—as well as population-specific factors in Chinese eye-bank donors and inherent differences between *in-vivo* physiology and post-mortem donor tissue. Although advanced age is associated with lower endothelial cell counts, increased polymorphism, impaired cellular function, and reduced stress tolerance—all of which may compromise graft survival (15)—age alone should not serve as an absolute exclusion criterion. Indeed, Greenwald et al. (16) reported that corneas from donors aged 76–80 years had graft suitability rates comparable to those from donors aged 71–75 years, with no significant differences in early postoperative outcomes. With rigorous quality control, corneas from elderly donors remain transplantable, thereby helping to alleviate global donor shortages.

As shown in **Supplementary Table S1**, donors who died of malignant tumors tended to be younger than those with chronic organ failure; all cause-of-death comparisons in the GLMM were therefore adjusted for donor age. Although donors with chronic organ failure exhibited significantly lower ECD than those who died of CCVDs, cause of death showed no overall independent effect in our model. Krohn et al. (12) proposed that chronic wasting conditions such as malignancy may accelerate endothelial loss through cachexia and catabolic metabolism, potentially interacting with age. However, we found no significant ECD difference between the malignant-tumor and chronic-organ-failure groups, a result that may be attributable to sample composition and the lack of stratification for tumor stage and disease duration. Accordingly, cause of death should not serve as a standalone exclusion metric and must be interpreted in conjunction with other donor indices. We did not identify an independent effect of DM on ECD—a finding that conflicts with some published results (7, 17). This discrepancy may arise from the predominantly mild-diabetes and short disease duration in our donor cohort. Larger-sample prospective studies with detailed DM subtyping, disease duration, and glycemic-control metrics are required for further validation.

Notably, numerous statistically significant inter-group differences in ECD were associated with median values well above the widely accepted clinical transplantation threshold of 2,000 cells/mm². This highlights the critical distinction between statistical significance and clinical relevance, suggesting that these risk factors should be interpreted as screening indicators rather than rigid exclusion criteria for donor corneas.

All corneas analyzed in this study were sourced from a Chinese eye-bank cohort, whose distinct demographic characteristics reflect the interplay of regional eye-bank development, clinical demand, and population health profiles, thereby conferring region-specific practical value to our findings. In China, cultural factors continue to influence public willingness toward corneal donation; despite a gradual increase in family consent, overall donation rates remain substantially lower than those in high-income countries, rendering donor corneal tissue a particularly scarce resource. Consequently, our findings offer meaningful implications for refining domestic donor-screening protocols and reducing unnecessary tissue wastage.

Globally, the principal procurement techniques used by eye banks include whole-globe enucleation (11) and *in-situ* sclerocorneal-button excision (4). This study adopted whole-globe enucleation. This approach offers several advantages: it maximally preserves corneal integrity, is technically straightforward, avoids complex on-site corneal dissection, minimizes the risk of tissue injury, and performs effectively under non-sterile procurement conditions, thereby reducing contamination risk. These benefits partly explain its widespread use in Chinese eye banks. Furthermore, scleral tissue obtained via whole-globe enucleation (18) has broad clinical utility in ocular reconstruction (19), glaucoma surgery (20, 21), vitreoretinal procedures (22), and ophthalmic plastic interventions (23).

Strengths of this study include its large sample size, comprehensive dataset, and use of GLMM to account for inter-eye clustering within individual donors, thereby enhancing the robustness of the results. In addition, *post-hoc* subgroup analyses clarified differences in endothelial cell density (ECD) across age and cause-of-death strata, supporting stratified donor screening practices.

Nevertheless, this study has several important limitations. First, as a single-center retrospective investigation, it cannot fully exclude selection bias. Diabetes mellitus was recorded as a binary variable in our registry; consequently, granular data on disease duration, HbA1c levels, antidiabetic therapy, and microvascular complications were unavailable. This coarse classification limits firm conclusions about the DM–ECD association and may partly explain the non-significant result.

Second, “chronic organ failure” is a composite registry diagnosis without further subgroup specification (e.g., recipients of renal replacement therapy); therefore, interpretations for this category warrant caution. Only seven donors had COPD, and they were classified within the chronic organ failure group, a sample too small for reliable subgroup analysis.

Third, two subgroups were small: donors with prior cataract surgery (n = 38, 2.6%) and those who died from traumatic accidents (n = 20, 1.4%). The latter yielded wide confidence intervals, indicating substantial statistical uncertainty. Although prior cataract surgery remained a strong predictor in the GLMM, effect sizes derived from these small subgroups should be interpreted cautiously and not overgeneralized. In addition, the borderline P-value for cancer in the full-variable model means that a weak independent cancer–ECD association cannot be definitively excluded.

Fourth, the DTP–ECD correlation was derived from cross-sectional eye-bank data and cannot be interpreted as evidence of longitudinal, causal endothelial decay over time. ECD was measured only after procurement, without a baseline assessment immediately post-mortem. Although the negative association was stable across the observed DTP range and quadratic sensitivity analysis revealed no obvious nonlinearity, the hourly effect magnitude should not be extrapolated to longer preservation periods, nor should it be interpreted purely as cold-storage-related physiological decay. Moreover, although enucleation was performed promptly after death, donor bodies were generally kept at room temperature before procurement. The duration of pre-enucleation temperature exposure was not documented; only post-enucleation transport at 2–8 °C was strictly controlled. This unmeasured confounder may partly account for the observed DTP–ECD relationship.

Finally, ECD was the only primary outcome examined. Although routine eye-bank screening systematically records endothelial-cell density, it does not archive morphological indices such as cell-size variation, polymegethism, and hexagonality. Therefore, future prospective studies incorporating comprehensive endothelial morphological assessment are required to validate our observations.

In summary, based on a large-scale corneal donor dataset from a Chinese eye-bank cohort, this study suggests that donor age, prior cataract surgery, and death-to-preservation time are key independent correlates of corneal endothelial cell density, with cause of death serving as a secondary predictive indicator. Given the relative scarcity of donor corneal tissue and the substantial burden of corneal blindness in China, the findings of this study provide targeted evidence for optimizing donor screening strategies and improving corneal resource allocation efficiency in domestic eye banks, which may facilitate standardized and refined corneal transplantation practice in China.

## Data Availability

The raw data supporting the conclusions of this article will be made available by the authors, without undue reservation.
