# Supplementary material for: Analysis of clinical factors affecting corneal endothelial cell density in eye bank donors
Source: Front Ophthalmol (Lausanne). 2026 Sep 16;6:1952825. doi: 10.3389/fopht.2026.1952825 (PMC13623622; doi:10.3389/fopht.2026.1952825)
Supplement: Supplementary file 1 [file DataSheet1.pdf]

SUPPLEMENTARY TABLE S1 Mean donor age stratified by different cause-of-death groups

Cause of death	N	Mean age(years)	Standard deviation
CCVDs	409	66.87	13.82
Malignant tumors	654	65.00	10.67
Traumatic accidents	20	54.50	15.23
Chronic organ failure	355	68.26	11.74

Note: Descriptive summary of donor age across different cause-of-death subgroups. No inferential statistical tests were conducted in this table; all group-level comparisons of ECD against cause of death were performed within the age-adjusted generalized linear mixed model.
